# Intact Susceptibility to Visual Illusions in Autistic Individuals

**DOI:** 10.1002/aur.70044

**Published:** 2025-04-21

**Authors:** Yarden Mazuz, Bat‐Sheva Hadad, Tzvi Ganel

**Affiliations:** ^1^ Department of Psychology Ben‐Gurion University of the Negev Beer‐Sheva Israel; ^2^ Department of Special Education and the Edmond J. Safra Brain Research Center University of Haifa Haifa Israel

**Keywords:** Autism, Perceptual priors, Size discrimination, Size perception, Visual Illusions

## Abstract

Altered sensory perception, a core characteristic of autism, has been attributed to attenuated use of stimuli context or prior information in perception. Reduced susceptibility to perceptual illusions was extensively used to support these accounts for autistic perception. However, empirical evidence has been inconsistent. The current study systematically investigated susceptibility to size illusions in autistic and non‐autistic individuals using a standardized psychophysical battery. Eighty‐one participants, 41 autistic and 40 non‐autistic individuals, completed the Ben‐Gurion University Test for Perceptual Illusions (BTPI), measuring susceptibility to the Ponzo, Ebbinghaus, and Height‐width illusions. The results demonstrate clear evidence for susceptibility to illusions in the perception of size both in the autistic and non‐autistic groups. No significant differences were found between groups in the magnitude of illusion on the perceived size, or on the perceptual resolutions of size (discrimination thresholds) in any of the illusory settings tested. The results challenge current theories suggesting reduced reliance on priors or enhanced sensory measurement in autism. Instead, using robust psychophysical methods, the study provides clear evidence for autistic people forming priors and using long‐term knowledge in perception.

## 1 Introduction

Autism spectrum disorder (ASD) is characterized by persistent challenges in social communication and interaction, restricted interests, and repetitive behavioral patterns (American Psychiatric Association 2013). Much of autism research has focused on social cognition and communication difficulties associated with the condition. However, the recent revision of the diagnostic criteria for autism has brought sensory processing into focus as another key domain of the autistic phenotype. This line of research has yielded mixed findings even for basic perceptual discrimination abilities, demonstrating heightened sensitivity (hypersensitivity), as well as reduced sensitivity (hyposensitivity) to the same perceptual aspects (Hadad and Yashar 2022). Several theories have been proposed to account for these perceptual alterations (see Robertson and Baron‐Cohen 2017, and Hadad and Yashar 2022, for reviews), and many of these theories have used reduced susceptibility to perceptual illusions as a central argument. However, little and mixed empirical evidence has been provided to support their strong claims. Here, we systematically test susceptibility to perceptual illusions in autistic and non‐autistic individuals using psychophysical tools within a new standardized battery of testing.

Dominant theories suggested that a centralized deficit in domain‐general cognitive processes underlies deficits in both sensory and social‐cognitive processing in autism. The “weak central coherence” hypothesis posits that the detail‐focused perceptual style in autism arises from deficits in extracting the global representation of a scene (Happé and Frith 2006). This has been supported by evidence showing enhanced processing of the local details of hierarchical visual patterns and of higher, more complex scenes (Behrmann et al. 2006; Robertson et al. 2012; Sasson 2006). Findings, however, have been inconclusive in determining whether weak global perception is indeed accompanying the local preference characterizing autistic perception (e.g., Chung and Son 2020; Hadad and Ziv 2015; Mottron et al. 2006).

Autistic individuals have also been posited to have perceptual representations in which bottom‐up sensory input is weighted more than top‐down predictions. This has been studied within the Bayesian framework, modeling perception as an integration of prior knowledge (top‐down processes) and sensory input (Ma et al. 2023). Bayesian accounts of autism have attributed altered perception to reduced priors (Pellicano and Burr 2012) or to enhanced measurement of sensory input (Brock 2012), with either of these accounts resulting in increased reliance on sensory information. However, findings failed to demonstrate clear deficits in the use of priors, nor in providing evidence for enhanced sensory measurement. Furthermore, several recent studies propose that autistic individuals mainly exhibit difficulties in properly updating their priors (Binur et al. 2022; Lieder et al. 2019; Vishne et al. 2021; Sapey‐Triomphe et al. 2021; Twito et al. 2024).

A valuable approach to test hypotheses derived from the perceptual Bayesian model is achieved by examining susceptibility to perceptual illusions. Such illusions occur in situations in which predictive processes largely override sensory information, and the magnitude of the illusion provides insights as to relative reliance on such predictive knowledge (Ma et al. 2023; Petzschner et al. 2015; Weiss et al. 2002). Previous research on potential differences between autistic and non‐autistic individuals in respect to their susceptibility to visual illusions has yielded mixed findings. Early studies, such as that performed by Happé in 1996, reported lower susceptibility among individuals with autism to six different visual illusions. However, subsequent research has yielded inconsistent results, with some studies reporting differences between autistic and non‐autistic individuals (Bölte et al. 2007; Mitchell et al. 2010), while others found no significant differences between the two populations (Binur et al. 2022; Hoy et al. 2004; Manning et al. 2017; Ropar and Mitchell 2001).

The idea that there are no significant differences between autistic and non‐autistic individuals in susceptibility to visual illusions has also gained support from studies focusing on autistic traits in non‐clinical populations. For example, Chouinard et al. (2016) employed a battery of visual illusions, including control tasks on basic visual functions, and found no differences in illusion susceptibility among non‐autistic individuals with varying levels of autistic traits. This pattern was true for 11 out of the 13 visual illusions tested, including the Ebbinghaus and the Ponzo illusions (Chouinard et al. 2016). While these findings are relevant to the current discussion on perceptual processing in autism, researchers have cautioned against extending findings from autistic traits in the general population to autistic individuals (Sasson and Bottema‐Beutel 2022). Therefore, we will now focus our discussion mainly on perceptual processing in autistic individuals.

Recently, Hadad and Yashar (2022) suggested that the discrepancy in findings on illusory effects in autism may stem not only from the heterogeneity within the ASD population but also from methodological issues in studies involving autistic individuals. They noted that studies that used established psychophysical methods have consistently reported no statistical differences in perceptual performance between autistic and non‐autistic individuals (e.g., Binur et al. 2022; Manning et al. 2017). Consistent with this suggestion, recent testing of priors' effects demonstrated typical use of prior knowledge in autism, both for illusory and non‐illusory settings, in particular when long‐term (naturally learned) knowledge is used as priors. This new line of research further suggests that differences between autistic and non‐autistic individuals may stem from atypical weighting of priors to changes in sensory noise (Binur et al. 2022; Sapey‐Triomphe et al. 2021), or from weaker updating of formed priors (Lieder et al. 2019; Twito et al. 2024).

The primary goal of the present study was to provide a thorough investigation of possible differences between autistic and non‐autistic individuals along perceptual mechanisms that underlie visual illusions. Group differences in the magnitude of the bias were tested for each illusion while measuring perceptual sensitivity to size, to control for potential differences in sensory measurement. To this purpose, we used a well‐established and standardized psychophysical battery that measures individual and group differences along the susceptibility to three fundamental visual illusions of size, each tapping a separate perceptual mechanism. We employed the Ben‐Gurion University Test for Perceptual Illusions (BTPI; Mazuz et al. 2023, 2024), a standardized battery typically administered online, to test susceptibility to visual illusions of size. The BTPI has shown robust reliability in measuring susceptibility to three visual illusions of size: the Ebbinghaus, the Ponzo, and the Height‐width illusions. In addition, the BTPI assesses perceptual resolution of size perception, indicated by the just noticeable difference (JND) within each illusion. Access to the BTPI is freely available through the following link: https://app.gorilla.sc/openmaterials/514763.

## 2 Materials and Methods

### 2.1 Participants

Eighty‐one participants were recruited for this study: 41 autistic individuals (32 males, 2 other; Mean age = 27.30, SD = 4.85, Range: 18–37), and 40 non‐autistic individuals (36 males, Mean age = 25.10, SD = 3.19, range: 15–35). The autistic participants were recruited from the local community and the “Beit Ekstein Center” for adults with communication difficulties. They all underwent the ADOS‐2 (Lord et al. 2000) assessment and only participants with a confirmed diagnosis of autism were included in the study. The ADOS scores averaged at 9.88 (minimum 7, maximum 16) for communication and social interaction. Autistic and non‐autistic participants were also measured on their intelligence measured by the Test of Nonverbal Intelligence (TONI4 test, Brown et al. 2010). Autistic participants were paid for their participation. For the non‐autistic group, participants were undergraduate psychology students from Ben‐Gurion University of the Negev who received course credit, as well as community members who were compensated with payment for their participation. All participants signed a consent form prior to the beginning the experiment. The experiment was approved by the BGU Department of Psychology Ethics Committee and by the Ethics Committee of the Faculty of Education at the University of Haifa #20/046.

IQ was assessed using the Test of Nonverbal Intelligence–Fourth Edition (TONI‐4), and there was no significant difference between the groups, *t*(64) = 1.17, *p* = 0.248 (autistic group: Mean = 101, SD = 12.10; non‐autistics group: Mean = 104, SD = 10.70). The Autism Spectrum Quotient (AQ; Baron‐Cohen et al. 2001) was used to measure autistic traits. AQ scores in the autistic group (Mean = 26.80, SD = 7.05) were significantly higher than those in the non‐autistic group (Mean = 15.10, SD = 4.57), *t*(65) = 7.57, *p* < 0.001. We note that due to technical difficulties, 14 participants from the non‐autistic group were not tested on the AQ and the IQ tests.

### 2.2 Procedure

**FIGURE 1:**
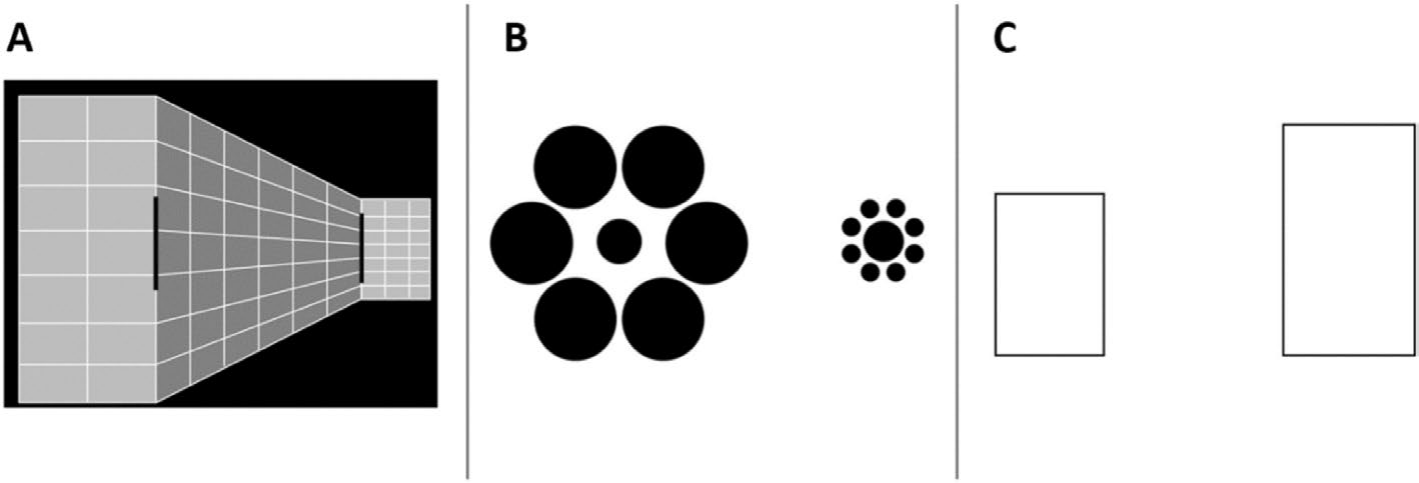
The stimuli used in the BTPI. Participants were instructed to choose the *longer* object in the Ponzo illusion (A); the larger *central* circle in the Ebbinghaus illusion (B); and the *wider* rectangle in the Height–width illusion (C).

The participants were tested using the BTPI battery (Mazuz et al. 2023), which utilizes a modified version of the method of constant stimuli, featuring three visual illusions: Ponzo, Ebbinghaus, and the Height‐width illusion (Figure 1). Typically, the BTPI is administered online. However, due to the demands of the present study, it was administered offline on a 14‐in. laptop computer for all participants. Each illusion was presented in a distinct block, following a predetermined sequence of Ponzo, Ebbinghaus, and then the Height‐width illusion. Within each block, stimuli were presented randomly, totaling 144 trials (12 repetitions of each of 12 standard‐reference combinations). Participants used the keys K and S on a standard keyboard to indicate their choice for the larger object in each trial, with K representing the right object and S the left.

In the Ponzo task, each trial commenced with a 1000 ms fixation cross and was succeeded by the presentation of stimuli. The stimuli were presented on the screen until the participant's response was recorded, with a maximum duration of 3000 ms. If no response was elicited within this timeframe, the subsequent trial commenced automatically. For the Ebbinghaus and Height‐width illusions, trials initiated with a 1000 ms fixation cross, followed by a 1000 ms display of the stimuli, after which participants presented with the ‘response display’ screen and typed their response. The total study duration was approximately 20 min. The BTPI demonstrated high test–retest reliability for the magnitude of the illusions and moderate reliability for JNDs (see Mazuz et al. 2023, 2024). We note that using a fixed 1000 ms presentation time for the Ebbinghaus and Height‐Width illusions was based on previous results (Bressan and Kramer 2021) and based on our own preliminary findings and experimentation during the development of the BTPI battery (Mazuz et al. 2023). This was done to prevent potential speed‐accuracy tradeoffs between presentation times and the susceptibility to these two illusions. For the Ponzo illusion, however, and based on our preliminary findings during the development of the BTPI (Mazuz et al. 2023), there were no speed‐accuracy tradeoffs. We therefore use a design in which the illusory display is presented until the participant's response (this design showed larger reliability compared to the fixed presentation time design, see Mazuz et al. 2023).

### 2.3 Data Analysis

Trials in which participants failed to respond within the specified time limit (3000 ms) were excluded from the analysis. Overall, the same proportion of trials (0.02%) were removed from the two groups' results. A sigmoid function $\frac{1}{1+e^{-\frac{x-A}{B}}}$ was fitted to each participant's data. The measures extracted from this analysis included the Point of Subjective Equality (PSE), Constant Error (CE), Just Noticeable Difference (JND), Reaction Time (RT), and the Goodness of Fit (GOF).

The CE, which indicates susceptibility to the illusion, was computed by subtracting the PSE (representing 50% “larger” responses) from the value of the standard stimulus. The JND, which reflects the perceptual resolution to size within the illusory context, was calculated as half the range between 25% and 75% of the function. Raw CE and JND scores were transformed to percentage scores for each participant, relative to the size of the standard stimulus.

RTs were recorded for each trial, and mean RTs were determined for each participant within each illusion. Outliers were identified and excluded if their RT deviated more than 3 standard deviations from the participant's average RT within each illusion. The measurement of RT in the Ponzo illusion task began from stimulus presentation until the participant's response, whereas for the Ebbinghaus and Height‐width illusion tasks, RT measurement began from the presentation of the response display, only after the 1000 ms stimulus exposure.

GOFs, denoting the fit of the sigmoid model in representing the observed data, were computed as the squared correlation coefficient between observed and predicted values. Only participants with GOF scores exceeding 0.7 for a specific illusory task were included for the final analysis (Mazuz et al. 2023). We conducted *t*‐tests to compare CEs and JNDs between autistic and non‐autistic individuals.

## 3 Results

Tables 1 and 2 display the average CEs and JNDs in each illusion for each group, including the one‐sample *t*‐test statistics indicating significant differences of the illusion effect from zero. The differences in sample sizes between the illusions are due to the exclusion of participants with goodness‐of‐fit (GOF) values smaller than 0.7. The results indicated that all CEs differed significantly from zero, providing evidence for illusion effects in both the autistic and non‐autistic groups.

**TABLE 1:** Average CEs for each illusion in each group (in percentages).

	Group	*N*	Mean	SD	Skewness		Kurtosis		*t*‐Test statistics	*p*
					Skewness	SE	Kurtosis	SE		
Ponzo	ASD	33	39.38	20.82	0.5369	0.409	−0.37991	0.798	10.87	< 0.001
	TD	36	35.57	21.31	0.3819	0.393	−0.35871	0.768	10.02	< 0.001
Ebbinghaus	ASD	32	46.29	15.19	0.3950	0.414	−0.00585	0.809	17.24	< 0.001
	TD	35	43.08	11.99	−0.4797	0.398	−0.03508	0.778	21.26	< 0.001
Height‐width	ASD	37	2.25	5.86	−0.0211	0.388	1.43696	0.759	2.33	0.025
	TD	38	1.77	4.17	−0.4859	0.383	0.32860	0.750	2.62	0.013

*Note*: The CEs represent the magnitude of the illusion. *t*‐test values indicate whether the illusion effect is significantly larger than 0.

Abbreviations: ASD, autistic group; TD, non‐autistic group.

**TABLE 2:** JNDs for each illusion in each group (in percentages).

	Group	*N*	Mean	SD	Skewness		Kurtosis	
					Skewness	SE	Kurtosis	SE
Ponzo	ASD	33	10.72	5.02	0.513	0.409	−0.656	0.798
	TD	36	11.38	5.83	1.188	0.393	1.220	0.768
Ebbinghaus	ASD	32	7.31	3.31	0.760	0.414	1.085	0.809
	TD	35	7.89	3.75	1.056	0.398	1.350	0.778
Height‐width	ASD	37	4.84	2.05	1.088	0.388	1.727	0.759
	TD	38	4.57	2.53	2.025	0.383	4.315	0.750

*Note*: The JNDs represent visual resolution for differences in size.

Abbreviations: ASD, autistic group; TD, non‐autistic group.

**FIGURE 2:**
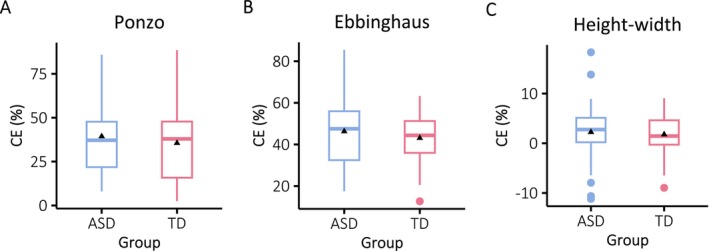
CEs (the magnitudes of the illusion, in percentage) for the Ponzo, Ebbinghaus and the Height‐width illusions for the autistic (ASD) and non‐autistic (TD) groups (from left to right, respectively). In all illusions, CEs were similar for the two groups. The box plots represent 50% of the central data (IQR), the black triangles are the means, and the insert lines represent the medians. The whiskers show the range of the data, they extend to 1.5 times the IQR from Q1 and Q3. The data points beyond this range are outliers and are plotted as points.

The *t*‐tests comparing the CE values between the autistic and non‐autistic groups revealed no significant differences for all illusions (Ponzo: *t*(67) = 0.75, *p* = 0.456; Ebbinghaus: *t*(65) = 0.96, *p* = 0.338; Height‐width: *t*(73) = 0.40, *p* = 0.686; see Figure 2). Furthermore, Bayesian independent *t*‐tests were used for the CEs to further test for possible differences between the groups. For the Ponzo illusion, the analysis yielded a Bayes Factor (BF01) of 3.18, suggesting that the null hypothesis is roughly three times more likely than the alternative hypothesis. Similarly, the Ebbinghaus illusion exhibited a Bayes Factor of 2.68, and the Height‐width illusion showed a Bayes Factor of 3.90, indicating that the null hypothesis is almost 4 times more likely than the alternative hypothesis.

To test if there is an overall trend for smaller susceptibility to illusions in the autistic compared to the non‐autistic group, we also performed a mixed ANOVA on the data across the three illusions. The independent variables were the group (autistic, non‐autistic) and the type of the illusion (Ebbinghaus, Height‐width, and Ponzo illusion). The main effect of group (autistic vs. non‐autistic participants) and the interaction between group and type of illusion were not significant (all Fs < 0). A significant main effect was found only for the type of the illusion (*F*(2,108) = 148.9, *p* < 0.001), which indicated differences along average susceptibilities (in percentage) to the three illusions.

We performed additional analyzes to test the idea that the susceptibility to illusions might have changed throughout experimental sessions, and that such changes could differ between autistic and non‐autistic individuals. To do so, we divided each category of trials (each of the 12 standard‐reference combinations) into two equal parts according to their order in the experimental session. We then calculated CEs separately for each part of the experiment. The results (shown in Table A1) showed that CEs were similar in the two parts.

**FIGURE 3:**
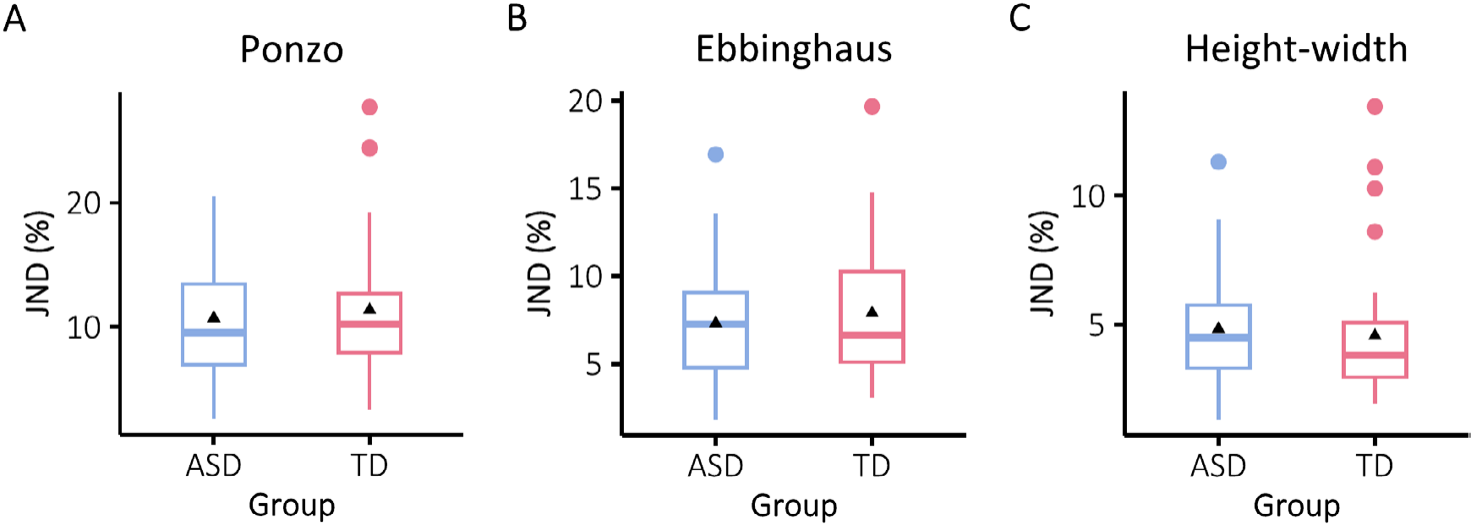
JNDs (sensitivity to size differences, in percentage) for the Ponzo, Ebbinghaus, and Height‐width illusions in the autistic (ASD) and non‐autistic (TD) groups (from left to right, respectively). In all illusions, JNDs were similar for the two groups.

In a similar manner, *t*‐tests for the JND scores between the autistic and non‐autistic groups did not yield any significant differences (Ponzo: *t*(67) = −0.50, *p* = 0.616; Ebbinghaus: *t*(65) = −0.67, *p* = 0.504; Height‐width: *t*(73) = 0.50, *p* = 0.615; see Figure 3). The overall pattern of results strongly suggests similar susceptibilities to the illusions and a similar sensitivity to size differences for the two groups. Unlike what has been previously assumed, we found evidence for the influence of the illusions in the autistic group that did not differ from that observed in the non‐autistic group.

Finally, we tested for possible patterns of correlation between CEs, JNDs, and AQs in the autistic and not autistic groups. For obvious statistical reasons, the relatively small sample sizes used in the current study (about 40 in each group) prevent making strong conclusions about the pattern of correlation within each group and about possible differences between the groups (compared to our previous studies in which sample sizes were considerably larger, see Mazuz et al. 2023, 2024). Although the pattern of correlations (see Table A2) seemed to be similar for the two groups, the results were quite noisy, which prevented drawing reliable conclusions as to possible patterns of correlations in the two groups. In addition, no significant correlations were found between AQ and the susceptibility to the illusions in each of the groups.

The RT analysis revealed differences between the groups in the Ebbinghaus and Height‐width illusions (Ebbinghaus: 355 ± 102 ms for the non‐autistic group; 510 ± 224 ms for the autistic group; t(65) = 3.71, *p* < 0.001; Height‐width: 335 ± 362 ms for the non‐autistic group; 479 ± 196 ms for the autistic group; t(73) = 3.81, *p* < 0.001). No statistical differences in RTs were found between the groups for the Ponzo illusion (1118 ± 123 ms for the non‐autistic group; 1142 ± 341 ms for the autistic group; *t*(67) = 0.28, *p* = 0.781). We note that the experimental design of the Ponzo illusion session was different from the one used for the Ebbinghaus and Height‐width illusions (Mazuz et al. 2023). Based on preliminary findings and experimentation in the process of developing the BTPI (Mazuz et al. 2023), we decided to use a design with limited presentation time (1000 ms) for the Ebbinghaus and height‐width illusions. Therefore, responses for these two illusions were recorded only when the stimulus disappeared (following its 1000 ms presentation). This means that the stimulus presentation time was fixed, and responses were made when the stimulus was not presented. Thus, longer response times for these two illusions do not necessarily reflect on response accuracy or on the susceptibility to the illusion (Mazuz et al. 2023). For the Ponzo illusion, however, and based on our preliminary findings during the development of the BTPI, we used a design in which the illusory display was presented until the participants responded. It is possible, therefore, that the slower responses of the autistic participants to the Ebbinghaus and Height‐width illusions, but not to the Ponzo illusion, could be attributed to the different task demands. In particular, it is possible that participants in the autistic group had difficulties in adjusting to a design in which they were asked to withhold their response until the illusory stimulus disappeared. Thus, we can speculate that they responded earlier than required during the stimulus presentation time, which necessitated pressing a key again at the response display screen, thereby prolonging the recorded RT. For the Ponzo illusion, however, in which they were not asked to do so, there were no significant differences in RTs between the groups. We note that the above account for the interaction between group and task demands remains speculative because, unfortunately, responses were not recorded during the initial 1000 ms presentation of the stimuli. Yet, to confirm that there were no correlations between response times and the susceptibility to illusions in the current study, we have conducted an additional correlation analysis within each group, that includes the susceptibility to illusions and the RTs for each of the illusions (Table A3). For both groups, no significant correlations were found between RTs and the susceptibility to the illusions.

## 4 Discussion

The primary objective of this study was to provide a standard, comprehensive investigation of possible differences between autistic and non‐autistic individuals in their susceptibility to perceptual illusions. To this purpose, we used a validated psychophysical battery that measures the susceptibility to three prominent visual illusions of size: the Ponzo, Ebbinghaus, and the Height‐width illusion. We provide clear indications for illusory effects on size perception in autism; no differences between autistic and non‐autistic individuals were found in susceptibility to the illusions, nor in resolution to size differences within the illusory contexts. Our findings provide strong evidence against the claims of reduced susceptibility to perceptual illusions, and more broadly, indicate the use of priors in autism.

Research on possible differences along the susceptibility to visual illusions between autistic and non‐autistic individuals has yielded inconsistent findings. Some studies indicated smaller or no illusory effects in autism (Bölte et al. 2007; Mitchell et al. 2010; Happé 1996). Yet, other studies have found intact susceptibility to visual illusion (Hoy et al. 2004; Ropar and Mitchell 2001). These discrepancies could be related to the nature of the task at hand. In particular, it is possible that indirect tools such as introspection and overt verbal reports obscure typical susceptibility to visual illusions in autistic individuals. When direct psychophysical measures were applied, which included a comprehensive psychometric evaluation of perceptual skills, the pattern of results reliably showed that autistic individuals are susceptible to visual illusions. For example, Ropar and Mitchell (1999) employed the method of adjustment in various visual illusions, including the Ponzo and Ebbinghaus illusions. Overall, autistic individuals showed susceptibility to these illusions. Typical susceptibility was also demonstrated for the Ponzo and Ebbinghaus displays in various psychophysics tasks such as 2AFC and perceptual adjudgments (Chouinard et al. 2013; Manning et al. 2017; Schwarzkopf et al. 2014). In Bayesian terms, the illusory bias is elicited by the observers' expectations about the sizes of objects within given contexts. The comparable effects of the bias found in the autistic group indicate that their perception is similarly affected by prior expectation and knowledge. Notably, we did not find differences in the precision of the sensory measurement either. JNDs, signifying the precision in discriminating size, were also comparable between the groups. This counters claims of more precise sensory measurement in autism underlining the seemingly attenuated use of priors (Brock 2012).

A potential general concern related to studies of illusory effects in autism is the age of the tested population. In particular, given that related previous studies focused both on adults (as we did in the current study) and on children, it is possible that the mixed pattern of results in previous studies could be accounted for by the age group of the tested population. Therefore, if age accounts for possible differences between ASD and controls, it could be expected that differences along the susceptibility to illusions would interact with the age group of the participants. A review of the relevant literature suggests otherwise. Previous evidence for smaller susceptibility to illusions in ASD was found both in children and adults. For example, Bölte et al. (2007) compared susceptibility for the Ebbinghaus and Ponzo illusions between adult individuals with autism (mean age 25.8) and between non‐autistic individuals and found smaller illusory effects in ASD for the two illusions. In a similar vein, Mitchell et al. (2010) showed smaller susceptibility to the Shepard illusion in adult individuals with autism (mean age: 21.1). Smaller susceptibility to illusions in ASD was also reported for children (mean age: 13; Happé 1996). As we previously discussed, there are mixed results to support the idea that autistic individuals show smaller susceptibility to illusions. A similar magnitude of the illusions in autistic and non‐autistic individuals was found for both children (Hoy et al. 2004; Manning et al. 2017; Ropar and Mitchell 2001) and adults (Binur et al. 2022). Our review of the literature suggests, therefore, that the mixed pattern of results in previous studies regarding differences along the susceptibility to illusions in autistic and non‐autistic individuals cannot be accounted for by differences along the age of the tested population.

It is possible that while autistic and non‐autistic individuals have similar priors, the two populations differ in how priors are adjusted in the context of illusions; Binur et al. (2022) investigated the idea of attenuated priors in autistic participants using a two‐alternative forced‐choice width discrimination task, employing the Height‐width illusion with Gaussian blur to manipulate sensory noise level. The results indicated that both groups perceived the illusion to the same extent under no blurring condition, yet non‐autistic participants displayed an increased bias with an increased sensory noise, while autistic participants exhibited consistent biases across different noise levels. These results suggest that while both populations perceive the Height‐width illusion to the same degree, autistic individuals demonstrate a distinct pattern of perceptual processing characterized by a non‐adaptive relative weighting of perceptual priors and sensory reliability.

Additional support for the idea that the use of priors in itself is not impaired in ASD highlights potential differences in updating and learning mechanisms between autistic and non‐autistic individuals. In particular, Lieder et al. (2019) and Vishne et al. (2021) suggested that perceptual differences in autism could be due to atypical learning mechanisms that influence how internal models are updated over time. A recently proposed mechanistic account suggests that altered neural connectivity might disrupt the balance between sensory input and prior expectations, engendering the inflexible update of priors in autism (Noel et al. 2024).

While the current results demonstrate a consistent pattern of findings across all the illusions in terms of the differences between autistic and non‐autistic individuals, it is interesting to note that previous literature has highlighted the absence of a common mechanism that underlies susceptibility to visual illusions (Coren et al. 1976; Cretenoud et al. 2019; Grzeczkowski et al. 2017; Mazuz et al. 2023; Hadad et al. 2019). Chouinard et al. (2013) tested the relation between autistic traits and the susceptibility to the Ebbinghaus, Ponzo, and Müller‐Lyer illusions. While no such relation was found for the Ebbinghaus and Ponzo illusions, the susceptibility to the Müller‐Lyer illusion decreased with higher AQ scores. These findings indicate that different visual illusions engage distinct perceptual mechanisms.

Interestingly, discrepancies emerge even within tasks that appear to assess the same general perceptual mechanism. In the current study, autistic participants demonstrated the illusory influence of height on perceived width for rectangles, suggesting they integrate object dimensions. However, for faces, autistic individuals often show weak recognition skills and a reduced inversion effect, which implies a shift from holistic processing to part‐based processing of faces (Hadad et al. 2019; Hartston et al. 2023). These differences suggest that perceptual alterations in autism might be specific to complex, socially relevant stimuli like faces. This aligns with the “eye avoidance” hypothesis, where autistic individuals avoid eye contact due to perceived social threat, hindering holistic face processing (Kliemann et al. 2012; Tanaka and Sung 2016). Furthermore, integrating features in faces might involve distinct processes compared to other non‐face objects. Therefore, focusing on these specific perceptual mechanisms, rather than a unified common principle for autism, could offer deeper insights.

The current findings of typical susceptibility to size illusions suggest that autistic perception incorporates the stimuli's context. However, prior studies reported a lack of a more general context‐dependent scaling in magnitude perception for autism, violating Weber's law (Hadad and Schwartz 2019) and showing reduced adaptation effects (e.g., Turi et al. 2015). This discrepancy may indicate that autism primarily affects basic sensory calibration, while higher perceptual computations influenced by context remain intact. This could also explain the irregular sensory sensitivities and behavior commonly observed in autistic individuals.

In summary, the current study employed a formal psychophysical tool and a standardized approach to compare the susceptibility to visual illusions between autistic and non‐autistic individuals. The results revealed no differences between the groups in their susceptibility to the illusions and in their resolution to size differences within the illusory contexts. The present approach underscores the importance of employing rigorous and standard psychophysical methodology when testing for potential differences between different populations. Our findings suggest that the perceptual mechanisms that underlie visual illusions of size in individuals with autism spectrum disorder are largely intact.

## Conflicts of Interest

The authors declare no conflicts of interest.

## Data Availability

The data that support the findings of this study are available from the corresponding author upon reasonable request.

## Appendix A

**TABLE A1:** Correlation matrices of the susceptibility to the illusions, JNDs, and AQ in each group.

ASD								
Correlation matrix								
		Ebbi	HW	Ponzo	JND% Ebbinghaus	JND% HW	JND% Ponzo	AQ
Ebbi	Pearson's r	—						
	df	—						
	*p*	—						
HW	Pearson's r	0.003	—					
	df	29	—					
	*p*	0.986	—					
Ponzo	Pearson's r	−0.044	0.177	—				
	df	23	28	—				
	*p*	0.836	0.349	—				
JND% Ebbinghaus	Pearson's r	0.448*	−0.188	−0.258	—			
	df	30	29	23	—			
	*p*	0.010	0.310	0.214	—			
JND% HW	Pearson's r	0.506**	−0.174	0.129	0.356*	—		
	df	29	35	28	29	—		
	*p*	0.004	0.304	0.496	0.049	—		
JND% Ponzo	Pearson's r	0.307	−0.010	0.162	0.247	0.292	—	
	df	23	28	31	23	28	—	
	*p*	0.135	0.958	0.367	0.235	0.118	—	
AQ	Pearson's r	−0.110	0.081	0.121	−0.250	0.018	0.144	—
	df	29	34	31	29	34	31	—
	*p*	0.555	0.640	0.502	0.176	0.916	0.425	—

Controls								
Correlation matrix								
		Ebbi	HW	Ponzo	JND% Ebbinghaus	JND% HW	JND% Ponzo	AQ
Ebbi	Pearson's r	—						
	df	—						
	*p*	—						
HW	Pearson's r	−0.022	—					
	df	64	—					
	*p*	0.861	—					
Ponzo	Pearson's r	−0.047	0.114	—				
	df	55	63	—				
	*p*	0.727	0.365	—				
JND% Ebbinghaus	Pearson's r	0.251	−0.109	−0.256	—			
	df	65	64	55	—			
	*p*	0.041	0.383	0.055	—			
JND% HW	Pearson's r	0.134	−0.250	0.155	0.491	—		
	df	64	73	63	64	—		
	*p*	0.284	0.030	0.216	< 0.001	—		
JND% Ponzo	Pearson's r	0.069	0.033	0.118	0.172	0.027	—	
	df	55	63	67	55	63	—	
	*p*	0.612	0.793	0.334	0.201	0.832	—	
AQ	Pearson's r	0.082	0.095	0.070	−0.105	0.134	0.081	—
	df	52	59	55	52	59	55	—
	*p*	0.558	0.465	0.606	0.450	0.304	0.547	—

*Note*: **p* < 0.05, ***p* < 0.01, ****p* < 0.001.

**TABLE A2:** Comparison of the first and second parts of the experimental session for each illusion.

Descriptives									
	Group	Period	*N*	Missing	Mean	Median	SD	Minimum	Maximum
CE._Ponzo	ASD	First part	30	12	39.68	37.47	19.40	8.17	85.33
		Second part	27	15	38.19	36.08	19.48	11.35	86.49
	TD	First part	31	10	32.52	33.78	23.68	−29.25	88.79
		Second part	31	10	36.55	38.29	22.21	2.41	88.94
CE._Ebbinghaus	ASD	First part	29	13	45.04	46.64	13.55	19.97	72.29
		Second part	29	13	45.15	46.22	14.77	15.17	76.02
	TD	First part	30	11	43.41	43.25	11.95	20.46	66.32
		Second part	36	5	43.02	44.05	12.15	18.48	75.08
CE._HW	ASD	First part	36	6	2.45	2.41	5.48	−12.19	16.54
		Second part	38	4	2.57	2.46	6.89	−12.49	20.54
	TD	First part	38	3	2.08	2.59	4.45	−8.42	10.26
		Second part	32	9	1.67	2.88	4.85	−10.02	9.82

ANOVA—CE._Ebbinghaus					
	Sum of Squares	df	Mean square	*F*	*p*
Group	108.386	1	108.386	0.63289	0.428
Period	0.599	1	0.599	0.00350	0.953
Group × Period	1.948	1	1.948	0.01137	0.915
Residuals	20550.723	120	171.256		

ANOVA—CE._HW					
	Sum of squares	df	Mean square	*F*	*p*
Period	0.796	1	0.796	0.0261	0.872
Group	14.405	1	14.405	0.4727	0.493
Period × Group	2.433	1	2.433	0.0798	0.778
Residuals	4266.749	140	30.477		

ANOVA—CE._Ponzo					
	Sum of squares	df	Mean square	*F*	*p*
Period	47.5	1	47.5	0.104	0.747
Group	573.9	1	573.9	1.260	0.264
Period × Group	225.8	1	225.8	0.496	0.483
Residuals	52399.5	115	455.6		

*Note*: Repeated‐measures ANOVAs.

**TABLE A3:** Correlation matrices between the susceptibility to illusions and RTs in each group.

ASD							
Correlation matrix							
		CE Ebbinghaus	CE HW	CE Ponzo	RT Ebbinghaus	RT HW	RT Ponzo
Ebbi	Pearson's r	—					
	df	—					
	*p*	—					
HW	Pearson's r	0.003	—				
	df	29	—				
	*p*	0.986	—				
Ponzo	Pearson's r	−0.044	0.177	—			
	df	23	28	—			
	*p*	0.836	0.349	—			
RT Ebbinghaus	Pearson's r	−0.054	−0.276	−0.287	—		
	df	30	29	23	—		
	*p*	0.767	0.133	0.164	—		
RT HW	Pearson's r	0.059	−0.217	−0.328	0.947***	—	
	df	29	35	28	29	—	
	*p*	0.751	0.197	0.076	< 0.001	—	
RT Ponzo	Pearson's r	−0.046	−0.396*	0.197	0.076	0.206	—
	df	23	28	31	23	28	—
	*p*	0.827	0.030	0.271	0.717	0.275	—

Controls							
Correlation matrix							
		CE Ebbinghaus	CE HW	CE Ponzo	RT Ebbinghaus	RT HW	RT Ponzo
CE Ebbinghaus	Pearson's r	—					
	df	—					
	*p*	—					
CE HW	Pearson's r	−0.054	—				
	df	33	—				
	*p*	0.757	—				
CE Ponzo	Pearson's r	−0.074	0.044	—			
	df	30	33	—			
	*p*	0.686	0.802	—			
RT Ebbinghaus	Pearson's r	−0.020	0.127	−0.066	—		
	df	33	33	30	—		
	*p*	0.907	0.468	0.718	—		
RT HW	Pearson's r	0.089	−0.054	0.029	0.723***	—	
	df	33	36	33	33	—	
	*p*	0.611	0.747	0.870	< 0.001	—	
RT Ponzo	Pearson's r	−0.170	−0.191	0.226	0.406*	0.218	—
	df	30	33	34	30	33	—
	*p*	0.353	0.273	0.186	0.021	0.209	—

*Note*: **p* < 0.05, ***p* < 0.01, ****p* < 0.001.
